# Pilot assessment of the sensitivity of the malaria thin film

**DOI:** 10.1186/1475-2875-7-22

**Published:** 2008-01-28

**Authors:** Colin Ohrt, Wendy Prudhomme O'Meara, Shon Remich, Peter McEvoy, Bernhards Ogutu, Ramadan Mtalib, James Sande Odera

**Affiliations:** 1 Walter Reed Army Institute of Research, Silver Spring, Maryland, USA; 2 Fogarty International Center, National Institutes of Health, Bethesda, Maryland, USA; 3 Malaria Diagnostics Centre for Excellence, Walter Reed Project; United States Army Medical Unit-Kenya, Centre for Clinical Research, Kenya Medical Research Institute, Kisumu, Kenya; 4 Armed Forces Institute of Pathology, Washington DC, USA

## Abstract

**Background:**

Malaria microscopy remains the reference standard for malaria diagnosis in clinical trials (drug and vaccine), new diagnostic evaluation, as well as in clinical care in much of the world today. It is known that microscopy is an imperfect gold standard, and that very low false positive rates can dramatically lower protective efficacy estimates in malaria prevention trials. Although new methods are now available, including malaria rapid diagnostic tests and PCR, neither is as yet validated in the clinical trial setting and both have limitations. Surprisingly, the sensitivity of thin smears is not well established and thin smears are not commonly used in the developing world.

**Methods:**

Malaria thick and thin films were collected in the lowlands of Western Kenya. All had density determined by four readings with two methods, as well as species identified. Thirty-six with low density parasitaemia had the thin smear read by five independent microscopists, two were expert and three were qualified. Microscopists read the entire thin film. For the first 10 parasites seen, they reported the species, appearance, time, field number, and red blood cells in the field. Total parasites, total fields, and total time to examine the smear were also recorded.

**Results:**

Median parasitaemia was 201 parasites/μl, mean 1,090 ± 2,195, range 6–11,124 parasites/μl for the 36 smears evaluated. The data revealed a density dependent increase in sensitivity, with 100% sensitivity achieved at >200 parasites/μl for experts and >500 parasites/μl for qualified readers. Thin film readings confirmed parasitaemia 74% of the time by experts, and 65% of the time for qualified microscopists. The 95^th ^percentile for time to detect parasitaemia was 15 minutes for experts, 17 minutes for qualified microscopists. This decreased to 4–10 minutes for experts at densities of > 200 parasites/μl. Additionally, substantial discordance for species identification was observed.

**Conclusion:**

The thin film is sensitive enough to be a useful tool to confirm malaria diagnosis in study subjects in some settings. Specificity of the thin film and its utility for confirming thick film or other diagnostic test results should be assessed further.

## Background

Light microscopy is the reference or "gold" standard for malaria diagnosis in clinical trials (drug and vaccine), new diagnostic evaluation, epidemiology studies, as well as in clinical management for much of the world today. It is known that microscopy is an imperfect gold standard [1-3]. The accuracy of microscopy relates to innate ability, training, experience, motivation, and laboratory resource.

Although new methods are now available, including malaria rapid diagnostic tests (RDTs) and PCR [1,3,4], neither are yet validated in the clinical trial setting. RDTs are coming into widespread clinical use in some parts of the world, and may become key to malaria management and control in many settings. However, currently available devices have limitations, such as batch-to-batch quality variation, species and density determination, persistent positivity, accuracy, and cost [4]. Also of concern with RDTs are reported false negative results in the presence of high parasitaemias [4]. The more affordable and robust HRPII-based RDTs only detect *Plasmodium falciparum*. Because of a variety of limitations of new methods, microscopy will continue to have a significant role in the clinical trial setting for several years to come, and as the reference standard to which new diagnostics devices are compared.

Serious problems resulting from false positive malaria smears in two Phase III clinical trials on two separate continents have been observed (unpublished observations). In recent publications [5-8], false positive smears appear to be a common problem with the reading of malaria thick films. Combinations of diagnostic methods are being actively pursued to minimize or eliminate this problem. Confirmation of positive smears with the thin film could be one strategy, especially if rapid confirmation of results is needed before study subject treatment.

The use of the malaria thin film varies widely in different settings. It is usually taught that the malaria thin film should be used for accurate species identification after malaria is identified on the thick film and for counting high density parasitaemia. However, in the developing world, thin films are rarely examined, and frequently not even made. Reasons cited by microscopists for not using the thin film include low sensitivity at low parasite densities, and that the time required is too demanding. On the other hand, laboratory technicians in the Western world rarely use the thick film, relying solely on the thin film for a definitive diagnosis. The rationale in this setting is that the thin film is felt to be easier to make and interpret.

In review of the literature, surprisingly, the sensitivity of the thin film relative to the thick film is not clearly defined. A standard reference states it is 30 fold less sensitive than the thick film [9], while a reference from 1917 reports ~1–4 fold less sensitive [10].

Classic work from Dowling and Shute reported that the thin film read for 10 minutes was similar to the thick smear read for 3 minutes [11]. Sensitivity of the thin film by parasite density was not reported in any of these references.

Dowling and Shute also reported that the thin film is much more efficient for identification of *Plasmodium ovale *[11]. They also deduced that 60–90% of parasites were lost during preparation of the thick smear.

This manuscript details a pilot experiment to begin to address the potential use of the thin smear in the clinical trial setting by assessing malaria smears with low parasitaemia using several readers to interpret both the thick film and thin films from the same smears. A second objective was to determine possible usefulness in the clinical or malaria control setting when RDTs are unavailable or suboptimal. The specificity of the thin film was not evaluated in this experiment and needs to be defined relative to and in combination with the thick smear.

## Methods

### Malaria smears

Malaria thick and thin films were collected as part of a screening effort to identify adult asymptomatic malaria carriers in Kombewa District, Kenya. The data reported for the thick films were collected during assessment of counting methods using white blood cells (WBC) versus a grid method [12].

Malaria smears were prepared from EDTA-preserved venipuncture blood within four hours of sample collection. Two microliters of blood were smeared to produce a thin film and 12 microliters of blood were spread in a circle with a 15 mm diameter on the same slide. Smears were air dried for 10–20 minutes and then the thin film fixed by dipping it only briefly into absolute methanol. The slides were individually stained with 3% Giemsa for one hour, then rinsed, and air dried.

Thirty-six slides were selected from a sample of 144 used in the counting study [12]. This sample was from a subgroup that were collected from asymptomatic semi-immune adults and therefore expected to be low. A WBC count was determined using a Coulter Counter for 29 of the 36 smears and was used to calculate parasitaemia using the WBC method. The seven smears without WBC counts had the mean WBC count of 5,200/μl substituted. Each malaria smear had its density estimated by two to four independent microscopists using a WBC or grid method. All density comparisons in this manuscript were based on a mean of the two independent 500 WBC estimates (parasites/500 WBC × WBCs/μl).

While counting parasites, each reader also reported species identified. Species from each of the four readings were compared to those from the thin film readings. Species identification was not a focus of the WBC versus grid experiment. Microscopists were instructed not to turn to the thin film for species identification.

### Microscopists

For the counting experiment, eight microscopists were trained during a "long" microscopy training workshop immediately prior to the study [7,12]. Slides were randomly assigned to each of the microscopists who were asked to read them using either the WBC method or the grid method. Not all microscopists participated equally, some performed as many as 25 readings, others as few as two. The same two readers did not perform counts using either method, but three did read both using the WBC and Grid methods. The readers consisted of three experts, and three qualified microscopists. Fifty-five percent of the four readings were performed by experts.

For the thin smear experiment, five microscopists who were available were selected. One expert reading thin smears (Expert 2) also read thick smears in the counting experiment above; the others were different individuals. In this experiment, each microscopist read every thin smear in a blinded fashion. Of the five readers, two were experts and three were qualified microscopists. Of the three qualified microscopists reading thin smears, one was newly trained and the other two had some difficulty on slide reading examinations.

### Counting methods

For the WBC method, parasites and WBCs were counted simultaneously on the thick film using a 100× oil-immersion objective, with the number of parasites recorded when 200 and 500 WBCs had been counted. One hundred high-powered fields were examined before a slide was declared negative. Participants were instructed to start reading the film in an area with at least eight WBCs. Counting was initiated when the first parasite was observed.

For the grid method, a 10 × 10 mm square grid divided into 100 smaller squares etched onto a glass circle was fit into the eyepiece of a microscope (Klarmann Rulings, Inc., Litchfield, NH). Parasites in the grid area in 100 high-powered fields were counted, with counting initiated in the first field. Parasite density was calculated based on volume per field. Based on the mean WBC count in the study population, 100 grids were approximately equivalent to 430 WBCs.

### Thin smear reading methods

By study specific procedure, microscopists were instructed to read the entire thin film. A form was provided to record the slide identification information and the following for each of the first 10 parasites identified: species, time to parasite, field number, and % typical. Percent typical was 100% if all characteristics were typical of a parasite, with progressively lower percentages if not all characteristics were correct. The final diagnosis, number of parasites seen, total fields, and time to read the smear were also recorded.

### Data analysis

Data were entered into, verified in, and calculations performed with Microsoft Office Excel 2003. Further statistical analyses were performed in and tables constructed with SPSS 14.0 for Windows. Means were compared with independent sample t-tests.

## Results

Thirty-six malaria thin films were blindly and independently read by five microscopists in order to better understand the utility of the thin smear in low density infections. These results were compared to density estimates from two independent readings on the thick film (differently designed initial experiment). Species reported from four independent readings by six microscopists reading thick films were also compared (thick smear readings for species include two more readings including the two grid readings). Median parasitaemia was 201 parasites/μl, mean 1,090 ± 2195, range 6–11,124 for the 36 smears evaluated.

### Sensitivity

Sensitivity of the thin film by parasite density is reported in Table 1, with breakdown by level of expertise in Table 2. The data reveals a density dependent increase in sensitivity, with 100% sensitivity achieved at > 200 parasites/μl for experts and > 500 parasites/μl for qualified readers. In the low density range studied, thin film readings confirmed parasitaemia 74% of the time by experts, and 65% of the time for qualified microscopists. 50–78% of the slides were positive by individual readers, and 92% of the slides were positive if any one of the five readers called it positive (Table 3). True negative smears were not included in the assessment; therefore, estimates of the specificity of the thin smear could not be made.

**Table 1:** Thin film sensitivity and other factors by parasite density

Sensitivity			Time to 1st Parasite	Field to 1st Parasite	% Typical 1st	RBC's Field 1st Parasite	Time to 2nd Parasite	Field to 2nd Parasite	% Typical 2nd	RBC's Field 2nd Parasite	Total Parasites	Total Fields	Total Time
											
Parasite	(95% CI)												
	
Densiy	n												
< 100	45	40% (25–55%)	6.5 ± 3.7* (12)**	155 ± 150	98 ± 6	195 ± 71	5.8 ± 5.4	245 ± 163	45 ± 52	103 ± 117	0.7 ± 1.3	475 ± 213	22 ± 10
101–200	50	46% (32–60%)	8.3 ± 7.7 (20)	194 ± 198	100 ± 1	199 ± 67	8.4 ± 4.4	268 ± 151	61 ± 50	146 ± 127	0.7 ± 1.1	455 ± 199	19 ± 8
201–300	15	87% (67–106%)	5.3 ± 3.4 (10)	139 ± 115	99 ± 3	188 ± 89	4.3 ± 2.0	85 ± 50	100 ± 1	162 ± 83	4.8 ± 3.5	490 ± 234	25 ± 12
301–500	15	93% (79–108%)	2.9 ± 1.7 (6)	69 ± 78	100 ± 1	237 ± 80	4.3 ± 3.3	59 ± 45	100 ± 1	257 ± 88	5.2 ± 3.8	475 ± 174	24 ± 13
501–1000	15	100% (NA)	4.2 ± 5.5 (20)	35 ± 32	98 ± 4	187 ± 48	3.3 ± 3.0	33 ± 28	99 ± 3	198 ± 59	8.1 ± 3.8	352 ± 214	24 ± 10
1001–2000	20	100% (NA)	2.7 ± 3.4 (11)	32 ± 39	99 ± 3	194 ± 78	3.8 ± 6.6	29 ± 33	98 ± 4	195 ± 79	9.2 ± 2.9	225 ± 134	17 ± 6
>2000	20	100% (NA)	4.2 ± 3.4 (11)	30 ± 59	98 ± 3	176 ± 57	3.2 ± 3.2	15 ± 15	99 ± 4	192 ± 68	10.4 ± 2.4	147 ± 82	25 ± 9

Total	180	68% (61–75%)	5.9 ± 5.5 (15)	113 ± 133	99 ± 3	203 ± 73	5.0 ± 4.8	97 ± 130	89 ± 29	192 ± 93	2.8 ± 4.0	412 ± 204	20 ± 10

**Table 2:** Thin film sensitivity and other factors by parasite density and reader level

Sensitivity				Time to 1st Parasite	Field to 1st Parasite	% Typical 1st	RBC's Field 1st Parasite	Time to 2nd Parasite	Field to 2nd Parasite	% Typical 2nd	RBC's Field 2nd Parasite	Total Parasites	Total Fields	Total Time
											
Parasite		(95% CI)												
		
Densiy		n												
*Expert*	< 100	18	44% (19–70%)	5.2 ± 4.3* (12)**	133 ± 174	100 ± 0	208 ± 95	4.8 ± 2.5	189 ± 137	50 ± 55	109 ± 100	1.0 ± 1.8	523 ± 202	22 ± 11
	101–200	20	55% (31–79%)	7.2 ± 6.6 (20)	272 ± 264	100 ± 0	217 ± 89	9.2 ± 3.9	237 ± 110	67 ± 52	158 ± 137	1.1 ± 1.5	572 ± 228	21 ± 7
	201–300	6	100% (NA)	4.7 ± 3.6 (10)	119 ± 83	98 ± 4	162 ± 61	4.2 ± 2.1	83 ± 67	100 ± 0	178 ± 77	6.2 ± 3.1	494 ± 239	26 ± 10
	301–500	6	100% (NA)	2.7 ± 1.6 (5)	61 ± 47	100 ± 0	214 ± 103	3.6 ± 2.8	56 ± 47	100 ± 0	267 ± 116	7.7 ± 3.4	560 ± 201	32 ± 13
	501–1000	6	100% (NA)	1.3 ± 1.0 (3)	29 ± 38	100 ± 0	186 ± 50	1.5 ± 0.5	27 ± 31	100 ± 0	195 ± 78	8.7 ± 3.9	276 ± 141	27 ± 11
	1001–2000	8	100% (NA)	1.1 ± 1.2 (4)	17 ± 33	100 ± 0	184 ± 101	2.0 ± 2.2	38 ± 46	100 ± 0	201 ± 113	10.0 ± 0.9	198 ± 168	19 ± 5
	>2000	8	100% (NA)	1.4 ± 1.2 (4)	7 ± 6	100 ± 0	157 ± 51	1.6 ± 1.0	11 ± 8	100 ± 0	183 ± 56	10.8 ± 1.0	96 ± 56	26 ± 5
*Qualified*	< 100	27	37% (18–57%)	7.6 ± 3.0 (11)	175 ± 133	96 ± 7	185 ± 48	6.7 ± 7.5	302 ± 179	40 ± 55	98 ± 144	0.5 ± 0.8	443 ± 218	22 ± 10
	101–200	30	40% (21–59%)	9.3 ± 8.8 (30)	123 ± 60	100 ± 1	182 ± 34	7.7 ± 5.1	294 ± 184	56 ± 52	136 ± 128	0.5 ± 0.7	376 ± 130	18 ± 9
	201–300	9	78% (44–112%)	5.9 ± 3.5 (10)	156 ± 141	99 ± 2	210 ± 107	4.3 ± 2.1	87 ± 34	99 ± 2	146 ± 93	3.9 ± 3.7	488 ± 245	24 ± 13
	301–500	9	89% (63–115%)	3.0 ± 1.9 (6)	75 ± 98	99 ± 2	254 ± 58	5.0 ± 3.8	62 ± 48	99 ± 2	247 ± 57	3.6 ± 3.2	418 ± 137	18 ± 9
	501–1000	9	100% (NA)	6.1 ± 6.5 (20)	39 ± 29	97 ± 5	189 ± 50	4.4 ± 3.5	37 ± 27	98 ± 4	200 ± 47	7.7 ± 3.9	403 ± 246	23 ± 10
	1001–2000	12	100% (NA)	3.7 ± 4.1 (15)	42 ± 41	98 ± 3 97	201 ± 62	5.1 ± 8.4	23 ± 19	97 ± 5	191 ± 46	8.7 ± 3.6	243 ± 111	15 ± 6
	>2000	12	100% (NA)	6.0 ± 3.2 (11)	45 ± 73	± 3	189 ± 59	4.3 ± 3.8	18 ± 18	98 ± 5	198 ± 77	10.2 ± 3.0	181 ± 81	25 ± 11

*Expert*	Total	72	74% (63–84%)	4.5 ± 5.2 (15)	120 ± 165	100 ± 2	206 ± 83	4.7 ± 4.4	96 ± 124	91 ± 28	203 ± 98	3.5 ± 4.2	495 ± 225	23 ± 10
*Qualified*	Total	108	64% (55–73%)	7.2 ± 5.6 (17)	106 ± 98	98 ± 4	200 ± 61	5.2 ± 5.2	97 ± 136	88 ± 30	182 ± 87	2.3 ± 3.9	356 ± 168	18 ± 9

*standard deviation **95th percentile

**Table 3:** Percentage of readings by species and reading on thin smear

**Result**	Expert 1	Expert 2	Qualified 1	Qualified 2	Qualified 3	Any One Reading*
**Positive**	69%	78%	69%	50%	75%	92%
**Pf**	76%	89%	88%	94%	89%	79%
**Pfm**	12%	0%	0%	0%	4%	12%
**Pfmo**	4%	0%	0%	0%	0%	3%
**Pfo**	4%	7%	8%	0%	4%	3%
**Pm**	4%	0%	4%	6%	4%	0%
**Po**	0%	4%	0%	0%	0%	3%

* positive or non-*P. falciparum *species if any reader identified

Pf = *P. falciparum*, Pfm = Pf &* P. malariae *mix, Pfmo = Pfm &* P. ovale *mix

Pfo = Pf + *P. ovale *mix, Pm = *P. malariae*, Po = *P. ovale*

The time for identification of the first and second parasites and field in which the parasite was observed is density dependent (Table 1 and Table 2). Expert microscopists identified the first parasite on average in 4.5 minutes, while the qualified took 7.2 minutes (p = 0.002). Expert microscopists also identified the first parasite as being more typical (p = 0.001). At > 300 parasites/μl, expert microscopists identified the first parasite in 1.1–2.7 minutes, with the 95^th ^percentile at 3–5 minutes. The qualified microscopists required more time with a mean time to first parasite taking 3–6.1 minutes, 95^th ^percentile 6–20 minutes in the same density range. Lower density ranges took more time for both groups. The red blood cells/field, total time to read, total fields, and total parasites seen under these conditions are also reported in Table 1 and Table 2.

### Species identification

Results by species and reading of the thick and thin film are presented in Table 3 and Table 4. Thin smear results by individual reader are presented in Table 3 and thick smear by group reading are presented in Table 4. On both thick and thin films, considerable variability in reporting appears to be present, and mixed infections appear to be common. Table 5 compares expert Reader 1 to the four thick film readings, more clearly illustrating the variability in reporting present.

**Table 4:** Percentage of readings by species and reading on thick smear

**Result**	Reading 1	Reading 2	Reading 3	Reading 4	Any One*
**Positive**	97%	100%	100%	100%	100%
**Pf**	80%	81%	86%	86%	64%
**Pfm**	17%	14%	11%	11%	28%
**Pfmo**	0%	0%	0%	0%	0%
**Pfo**	0%	3%	3%	0%	6%
**Pm**	3%	0%	0%	3%	0%
**Po**	0%	3%	0%	0%	3%

* positive or non-*P. falciparum *species if any reader identified

Pf = *P. falciparum*, Pfm = Pf &* P. malariae *mix, Pfmo = Pfm &* P. ovale *mix

Pfo = Pf + *P. ovale *mix, Pm = *P. malariae*, Po = *P. ovale*

**Table 5:** Thin smear species results from Expert 1 compared with four thick smear readings

**Thin Film**			**Thick Film**		
	Slide No.	Reading 1	Reading 2	Reading 3	Reading 4

**Negative**	1	Neg	Pf	Pf	Pf
	2	Pf	Pf	Pf	Pf
	3	Pf	Pf	Pf	Pf
	4	Pf	Po	Pf	Pf
	5	Pm	Pf	Pf	Pf & Pm
	6	Pf	Pf	Pf	Pf
	7	Pf	Pf	Pf	Pf
	8	Pf & Pm	Pf	Pf	Pf
	9	Pf	Pf	Pf	Pf
	10	Pf	Pf	Pf	Pf
	11	Pf	Pf	Pf	Pf
**Pf**	1	Pf	Pf	Pf	Pf
	2	Pf	Pf & Pm	Pf	Pf
	3	Pf	Pf	Pf	Pf
	4	Pf & Pm	Pf	Pf	Pf
	5	Pf	Pf	Pf	Pf
	6	Pf	Pf	Pf	Pf
	7	Pf	Pf	Pf	Pf
	8	Pf	Pf	Pf	Pf
	9	Pf	Pf	Pf	Pf
	10	Pf	Pf	Pf & Po	Pf
	11	Pf	Pf	Pf	Pf
	12	Pf	Pf & Pm	Pf	Pf & Pm
	13	Pf	Pf	Pf	Pf
	14	Pf	Pf	Pf	Pf
	15	Pf	Pf	Pf	Pf
	16	Pf	Pf	Pf	Pf
	17	Pf	Pf	Pf	Pf
	18	Pf	Pf	Pf	Pf
	19	Pf	Pf	Pf	Pf
**Pfm**	1	Pf & Pm	Pf & Pm	Pf	Pm
	2	Pf	Pf	Pf & Pm	Pf
	3	Pf & Pm	Pf & Pm	Pf & Pm	Pf
**Pfmo**	1	Pf & Pm	Pf & Pm	Pf & Pm	Pf & Pm
**Pfo**	1	Pf	Pf & Po	Pf	Pf
**Pm**	1	Pf & Pm	Pf	Pf & Pm	Pf & Pm

Pf = *P. falciparum*, Pfm = Pf &*P. malariae *mix, Pfmo = Pfm &*P. ovale *mix

Pfo = Pf + *P. ovale *mix, Pm = *P. malariae*, Po = *P. ovale*

Most of the discordant thick smear results were due to differing reports of mixed infections (Tables 3, 4, and 5). From the four independent readings on thick films, 36% were discordant (at least one of the four readings did not agree). The discordance resulted from the following: 25% mixed versus not, 6% mixed versus not and pure species disagreement, 3% pure species disagreement, and 3% positive versus not. The WBC (reading 1 and 2) and grid (reading 3 and 4) methods showed similar findings.

Most of the discordant thin smear results were due to differing reports of the presence of parasites (Tables 3 and 5). From the five independent readings on thin films of the same slides, 53% were discordant (at least one of the five readings did not agree). The discordance resulted from the following: 3% mixed versus not, 3% mixed versus not and pure species disagreement, 3% positive versus not and pure species disagreement, 3% positive versus not, mixed versus not, and pure species disagreement, and 42% positive versus not. Of the two experts reading thin films, 33% were discordant. The discordance resulted from the following: 6% mixed versus not, 3% mixed versus not and pure species disagreement, 3% positive versus not and pure species disagreement, and 22% positive versus not.

For the densities of infections examined in this experiment and using Expert 1 for the thin smear readings as the gold standard, the thin smear added *P. ovale *to the diagnosis in one case (Table 5). It confirmed the presence of mixed infection in three of four cases where mixed infection was identified by at least two of the other readers. It confirmed the presence of *Plasmodium malariae *in four of the six cases where this species was identified by at least two of the readers.

## Discussion

In this study, the potential utility of the thin film was explored. The intent was to determine at what parasite density it is appropriate to confirm parasites and species in the thin film when the thick film is read as positive. False positive thick smears are a problem [5-8] and very low rates of false positive malaria smears can dramatically impact efficacy estimates in malaria prevention trials [13]. Rapid verification of results is needed before study subjects are treated and removed from trials, and methods must be acceptable to regulatory authorities. Use of the thin smear to verify thick smear results is a possible tool, as are RDTs or a repeat thick film. PCR is not practical in most settings when treatment decisions must be made within hours, but can potentially be used later to help confirm malaria, malaria species, and if a new infection is a treatment failure or not.

If the results of this report are confirmed, the thin smear can be expected to be positive 100% of the time if parasitaemia are > 500 parasites/μl as a single reading by any qualified microscopist, and at > 200 parasites/μl with experts. The time required for experts to identify a 100% typical parasite on the thin film in this experiment was substantially less than that for less experienced microscopists. Parasite densities are often low in malaria prevention trial failures and in treatment trial failures, limiting the ability to detect parasites with a single reading of the thin film. Historical parasite density data was available from confirmed parasitaemia from two malaria prevention trials using weekly routine screening and microscopy at the time malaria symptoms developed. In the first population of non-immune study subjects [14], 95%, 86%, and 71% of *P. falciparum *infections were above 100, 200, and 300 parasites/μl, respectively, compared to 83%, 54%, and 30% of *Plasmodium vivax *infections. In a semi-immune adult population in Kenya, 60%, 54%, and 30% of *P. falciparum *parasitaemia were above 100, 200, and 300 parasites/μl, respectively (unpublished data). In malaria prevention trials, thousands of slides are often collected with only a very small percentage being positive. A procedure should be implemented that microscopists report the result of the thick and thin smear always in this setting. Based on probability of being positive at a given density, quality assurance personnel and the Principal Investigator could use the thin smear as in internal control for monitoring results and interpreting the data. They could also help with study subject management decisions.

One would think that false positive malaria smears are only reported as extremely low densities. However, for 81 false positive smears read from true negative smears in a counting examination, the mean (median) count was 591 (300) parasites/μl, with 66% > 200 and 32% > 500 parasites/μl (unpublished observations). The 24 microscopists from Kenyan research organizations had among the lowest reported densities for false positives. Their 37 false positive readings had 62% > 200 and 22% > 500 parasites/μl.

Specificity of the thin film was not assessed in this experiment, nor has it been reported in the literature. It is anticipated that it will be substantially higher than with thick films, as parasite morphology is clearer and parasites are identified within red blood cells on the thin film. Specificity must be confirmed by appropriately designed research studies assessing microscopists of varying levels of expertise if this tool is to be used to support clinical trials.

If these pilot data are confirmed and specificity of the thin film is reported to be very high, it is recommend the thin smear result be routinely recorded to verify malaria smears are truly positive for slides that will represent critical clinical trial endpoints (prophylaxis and treatment failure). These should be read by expert microscopists. Depending on human resource available, time could be limited to 10 minutes per thin film for experts to identify at least one parasite. Principal investigators will be able to use parasite density as a guide as to whether the results should be expected to be positive. Alternatively, strategies to have more than one reader, more than one thin film, or repeat sampling could be used for confirmation. Results from both the thick and thin film should always be permanently documented with high quality photographic images.

An additional potential use of the thin film would be to train microscopists to turn to the thin film routinely at densities when it is expected to be positive. Based on these data, this would be at 5 or 8 parasites/200 WBCs (~200 or 500 parasites/μl) to confirm a positive. It would make sense to do this in any setting when the microscopist is unsure of the result (e.g. < 100% typical appearance).

Alternative diagnostic procedures should be considered, but need to have sensitivity and specificity estimates for the intended use verified, and ideally should be approved by the appropriate regulatory authority. The exact strategy should always be acceptable to the appropriate regulatory authorities for new malaria compounds or vaccines under development, and results from each reading or methods should be captured and reported.

PCR was not performed on the samples studied in this experiment. PCR may have helped clarify the actual species present. However, PCR results also vary by method used, and from laboratory to laboratory (unpublished observations). Future experiments should include the use of PCR using different methods and different laboratories. PCR should be validated so that it can be used as a primary endpoint in clinical trials.

RDTs were also not included in this experiment. Sensitivity and specificity estimates vary widely in the literature for the same RDT [4]. HRPII-based RDTs may be problematic in treatment trials because of persistence of positivity, may not be substantially more sensitive than a thin film, are not quantitative, and only identify *P. falciparum*. The specificity of these devices also needs to be considered, as well as regulatory approval and batch-to-batch variability. RDTs are found to be very useful for screening in order to rapidly help identify study subjects that may qualify for a given study, but have not yet assessed their potential for determination of primary endpoints in clinical trials.

Cross contamination causing false positive malaria smears clearly occurs with batch staining [15-17]. If single staining cannot be used, the thin smear will separate contamination from true positive smears. Parasites will be visualized in the red blood if a smear is truly positive.

In terms of species identification, the only clear advantage of the thin smear over the thick film appears to be identification of *P. ovale *in experienced hands [11]. *P. ovale *does not always have the "red zone" on the thick film, and if not, it is commonly confused with *P. malariae*. There appeared to be one of 36 cases in this experiment where *P. ovale *was added to the diagnosis. Mixed infections were commonly not confirmed, probably due to low density of the additional species. PCR in future studies may help confirm if this is indeed true.

Initial evaluation has revealed that some laboratory technicians are not achieving adequate sensitivity and specificity using thick malaria smears following two weeks of training [7]. In the Western world, only thin smears are routinely used reportedly because they are easier to prepare correctly and interpret. Whether the use of the thin smear in health care settings in the developing world would be beneficial should be critically studied. They may have acceptable ease of use, sufficient accuracy, and be more cost effective than RDTs in some settings. A key advantage of thin films over RDTs is the ability to estimate parasite density and define specific species.

## Conclusion

The data revealed a density dependent increase in sensitivity, with 100% sensitivity achieved at > 200 parasites/μl for experts and > 500 parasites/μl for qualified readers. Substantial discordance for species identification was observed. The thin film is may be additional tool to confirm a malaria diagnosis. Specificity of the thin film and its utility for confirming thick film results should be assessed further.

## Abbreviations

RDTs: Rapid Diagnostic Tests

HRPII: Histidine-Rich Protein II

EDTA: Ethylenediaminetetraacetic Acid

WBC: White Blood Cell

## Authors' contributions

CO designed this experiment, coordinated data collection and verification, performed analysis, interpreted the data, and wrote the manuscript. WPO was responsible for the thick smear counts and species determination. SR was responsible for day-to-day management of staff and provided the source slides. PM helped to conceive the study and revised the manuscript critically for substantial intellectual content. BO is Director of the Malaria Diagnostics Center of Excellence. He revised the manuscript critically for substantial intellectual content. RM and JSO were the expert microscopists interpreting the slides and final data. All authors read and approved the manuscript.
